# Negative Feedback and Transcriptional Overshooting in a Regulatory Network for Horizontal Gene Transfer

**DOI:** 10.1371/journal.pgen.1004171

**Published:** 2014-02-27

**Authors:** Raul Fernandez-Lopez, Irene del Campo, Carlos Revilla, Ana Cuevas, Fernando de la Cruz

**Affiliations:** Instituto de Biomedicina y Biotecnologia de Cantabria (IBBTEC), Santander, Spain; Université Paris Descartes, INSERM U1001, France

## Abstract

Horizontal gene transfer (HGT) is a major force driving bacterial evolution. Because of their ability to cross inter-species barriers, bacterial plasmids are essential agents for HGT. This ability, however, poses specific requisites on plasmid physiology, in particular the need to overcome a multilevel selection process with opposing demands. We analyzed the transcriptional network of plasmid R388, one of the most promiscuous plasmids in *Proteobacteria*. Transcriptional analysis by fluorescence expression profiling and quantitative PCR revealed a regulatory network controlled by six transcriptional repressors. The regulatory network relied on strong promoters, which were tightly repressed in negative feedback loops. Computational simulations and theoretical analysis indicated that this architecture would show a transcriptional burst after plasmid conjugation, linking the magnitude of the feedback gain with the intensity of the transcriptional burst. Experimental analysis showed that transcriptional overshooting occurred when the plasmid invaded a new population of susceptible cells. We propose that transcriptional overshooting allows genome rebooting after horizontal gene transfer, and might have an adaptive role in overcoming the opposing demands of multilevel selection.

## Author Summary

In the environment, bacteria often evolve by the acquisition of new genes from different species. Plasmids are small DNA molecules that mediate horizontal gene transfer in bacteria, thus they are fundamental agents for the spread of antibiotic resistances. Plasmids replicate inside the bacterial cytoplasm, and propagate infectiously by contact. Plasmids control these two ways of multiplication, but like many other symbionts they suffer from a tradeoff. If plasmids become very infective, they can spread fast and successfully, but this damages the bacterial hosts they depend upon. If, on the contrary, plasmids become very mild, the host is able to grow better but the ability of plasmids to infect new hosts is hampered. We have studied the regulatory mechanisms plasmids use to overcome this paradox. We discovered that negative feedback, a regulatory motif ubiquitous in the plasmid network, allows transient activation of plasmid functions immediately after plasmids invade a new host. This might be an adaptive strategy for plasmids to be highly infective without damaging their hosts, and it illustrates a natural mechanism for DNA transplantation that could be implemented in synthetic genomic transplants.

## Introduction

Horizontal gene transfer (HGT) is ubiquitous in bacteria. Because its important role in bacterial adaptation, HGT has been traditionally compared to sexual reproduction in higher eukaryotes. In bacteria, however, HGT is not mediated by specific intracellular mechanisms, but it is the byproduct of the pervasive movement of a myriad of mobile genetic elements. These include transposons, phages, ICEs and, most notably, plasmids [1]. Among them, broad host range (BHR) plasmids of *Proteobacteria* stand out because of their ability to colonize a wide range of bacterial species. This ability makes BHR plasmids efficient shuttles for HGT, clearly illustrated in the last decades by their leading role in the spread of antibiotic resistance genes among microbial populations [2].

Bacterial plasmids are agents for HGT, but they themselves are genetic replicons with their own, idiosyncratic, evolutionary history [3], [4]. Plasmid fitness depends on two basic physiological functions: maintenance within the bacterial host and transfer into new hosts; functions that are encoded in the plasmid genome. However, since plasmids can only exist inside a bacterial cell, their fitness is also host dependent. Plasmids impose a burden on host fitness [5], [6], [7], [8], which is dependent on the collective effect of the plasmid population within a given cell. The overall fitness of a plasmid replicon therefore depends not only on its own phenotype, but also on the phenotype of other co-residing plasmid copies. This dependency on the group phenotype puts plasmids under multilevel selection [9]. Multilevel selection forces plasmids to confront a paradoxical situation. Competition between plasmid copies within a given cell favors plasmids with higher copy number, superior partition systems and higher transfer rates [9]. However, these processes come to a cost, since plasmid consumption of resources imposes a metabolic burden that hampers host fitness. Competition between plasmid-containing cells, on the contrary, selects for plasmids that minimize the burden imposed onto the host. Both selection levels are thus intrinsically in conflict, and an adequate regulation of gene expression becomes essential to ensure plasmid survival [10].

Transcriptional regulation is common in plasmids, and virtually all functions in plasmid physiology have been found to be under transcriptional control [11]. In some cases, this control is exerted in sophisticated and apparently redundant layers. For example, plasmid R1 replication is controlled simultaneously by an antisense RNA and a transcriptional repressor [12] [13]. In other cases, like in the broad host range plasmid RP4, transcriptional regulation is organized under a global network that coordinates all functions in the plasmid's physiology [14]. Unfortunately, despite our knowledge of the molecular biology of transcriptional regulation, our understanding of the signals that plasmid regulatory circuits respond to is scarce. Plasmids from *Gram*+ bacteria regulate conjugation according to external cues about the abundance of possible receptors. These cues are communicated in the form of specific pheromones [15]. Such systems are generally not found in plasmids from *Proteobacteria*, with the remarkable exception of Ti-like plasmids from *Alpha-Proteobacteria*, which monitor external conditions via a quorum sensing mechanism[16] [17]. Apart from these and a few other cases, the input information that plasmid regulatory circuits monitor remains elusive.

Trying to understand the fundamental principles of plasmid transcriptional control, we analyzed the regulatory network of plasmid R388. Plasmid R388 is the smallest BHR conjugative plasmid found in *Proteobacteria* [3]. It shows an extensive host range, which overlaps with that of plasmid RP4, another model BHR plasmid [18]. Remarkably, plasmid RP4 is phylogenetically unrelated to plasmid R388 [3]. This situation allowed us to compare two plasmid networks that evolved independently, but under similar selective constraints. Using fluorescence expression profiling and quantitative PCR, we found a global regulatory network that controlled plasmid R388 transcription. Unlike the archetypical plasmids from *Gram*+ bacteria or Ti-like plasmids, the network seemed to be unresponsive to environmental changes, or quorum signals. The network was based on a basic regulatory motif: a strong promoter controlled by a tight negative feedback loop (NFL). We show, computationally and experimentally, that this architecture induces transcriptional overshooting after horizontal transfer of the plasmid.

## Results

**Figure 1:**
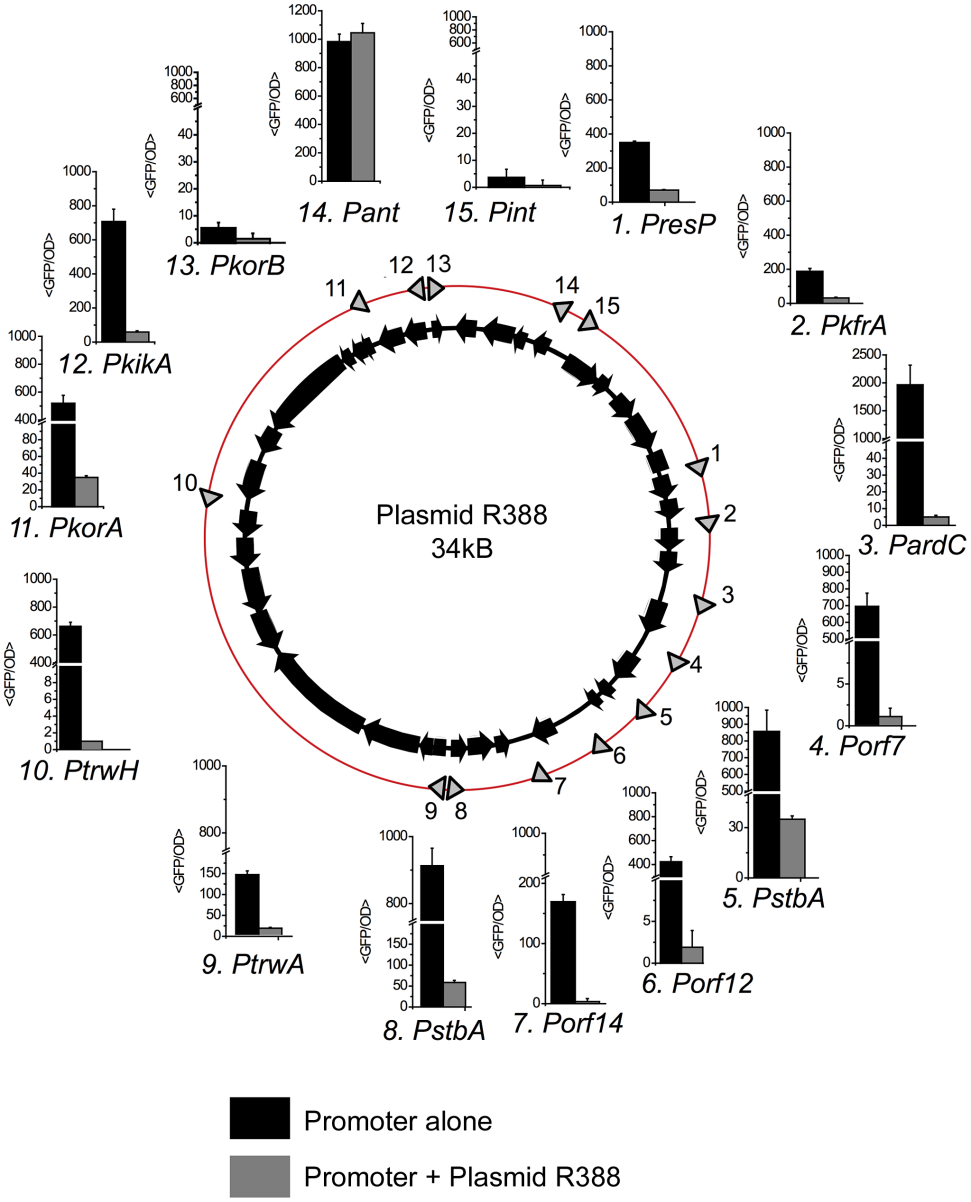
Promoters in plasmid R388. The figure shows the location and transcriptional activity of promoters detected in plasmid R388 genome. The location of each promoter is indicated by an arrow on the red circle. Each promoter receives the name of the first gene encoded in the transcriptional unit. Bar charts indicate the expression levels when the promoter activity was measured alone (black columns) or in cells that also contained plasmid R388 (grey columns). The expression levels (<GFP/OD>) represent the average GFP/OD (×10^2^) level achieved by cells growing in exponential phase. Each column represents the average and standard deviation of at least five independent experiments.

Intergenic regions in plasmid R388 DNA were PCR-amplified and cloned in the low copy number reporter vector pUA66 [19] to drive transcription of *gfpmut2* after a strong ribosomal binding site. Out of the 19 intergenic regions cloned, 15 showed transcriptional activities at least two-fold higher than the promoter-less vector, and were considered to contain a promoter (Figure 1). To compare the transcriptional strength of these promoters against a known standard, the activity of **P** *araBAD* was measured at different arabinose concentrations. **P** *araBAD* promoter reached 10^5^ GFP/OD units at maximal induction, and 13 out of 15 R388 promoters showed levels similar to this value (Figure 1, Supporting Table S1). Therefore R388 promoters, when assayed in the absence of the plasmid network, have strong transcriptional activities.

Transcriptional activity decreased sharply when the promoters were assayed in cells that also contained plasmid R388 (Figure 1, Supporting Table S1). The repression fold exerted by the plasmid ranged from 5 (**P** *resP*) to more than 500 fold (**P** *orf7*). Six promoters (**P** *ardC*, **P** *orf14*, **P** *orf12*, **P** *korB*, **P** *trwH*, **P** *orf7*) dropped to levels close to background, and another six (**P** *resP*, **P** *kfrA*, **P** *ssB*, **P** *stbA*, **P** *korA*, **P** *kikA*) showed values similar to those of repressed **P** *lacZ* (1*10^3^ GFP/OD). The only promoters that showed no changes in the presence of plasmid R388 were **P** *int* and **P** *ant*. Interestingly, these promoters do not belong to the plasmid backbone: they are part of the In-3 integron platform, which incorporated recently, in evolutionary terms, into the plasmid genetic structure [20]. Therefore, when the full regulatory network was present, all promoters from the plasmid backbone were repressed, and kept at levels lower or similar to LacI-repressed **P** *lacZ*.

**Figure 2:**
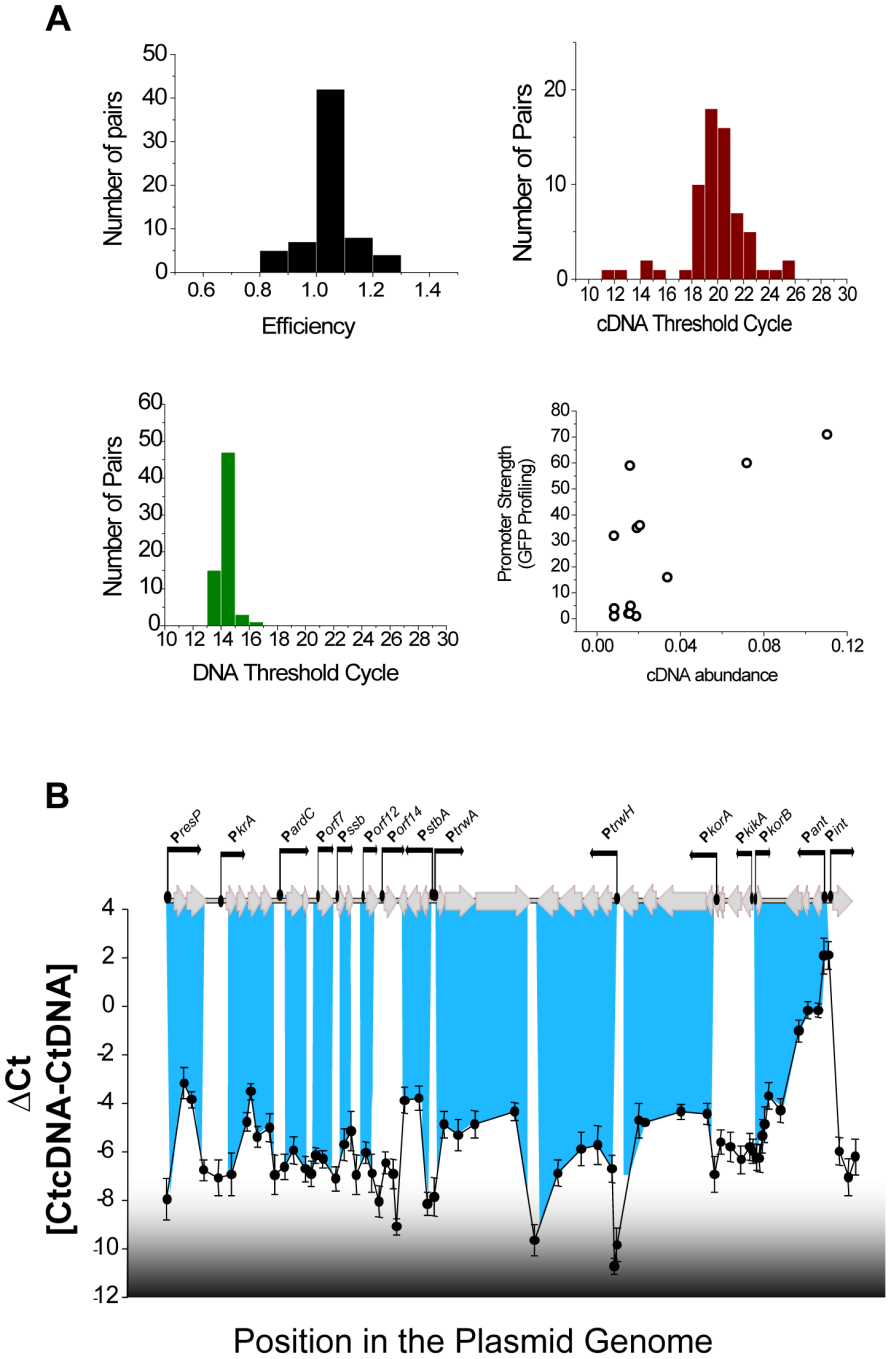
Transcriptional units in plasmid R388. (**A**) Statistics of the 66 primer pairs used to measure transcriptional levels in plasmid R388 (Upper right) Histogram showing the efficiency (calculated as indicated in materials and methods) of the primer pairs. (Upper right) Histogram showing the Ct obtained in qPCR amplifications from plasmid cDNA. (Lower left) Histogram showing the Ct obtained in qPCR amplifications from purified plasmid DNA. (Lower right) Scatter plot showing the relationship between the promoter activity (obtained from figure 1, in GFP/OD unit on the y axis) and the mRNA levels measured by RT-qPCR (ΔCt, x axis). (**B**) Transcriptional landscape of plasmid R388. The graph shows the relative abundance of mRNA, indicated as ΔCt = CtcDNA-CtDNA) along the plasmid genome. Each unit in the y axis corresponds to a 2 fold increase in mRNA. Peaks correspond to highly transcribed regions and valleys correspond to non-transcribed regions. The highlighted blue lines indicate the overlapping of the transcriptional units and the plasmid promoters identified in figure 1.

To determine the transcriptional units of the plasmid, mRNA levels during exponential growth were analysed by RT-qPCR. Relative mRNA abundance was measured using a set of 66 primer pairs, designed to cover the entire plasmid genome. From these 66 primer pairs, 60 showed efficiencies in the interval 0.9<E<1.2 (Figure 2A, left upper panel) and were considered suitable for quantification. mRNA was extracted from cells growing in rich media at mid-exponential phase, and retrotranscribed into cDNA as described in Materials and Methods. Using 300 ng total RNA, we obtained an average threshold cycle (Ct) of 19.9 with cv = 0.12. Results for each primer pair were normalized measuring the Ct corresponding to 5 ng of purified plasmid DNA. Results (Figure 2A, left lower panel), showed a tight distribution with an average Ct of 14.2 and cv = 0.034. Relative abundances of mRNAs were expressed as ΔCt = CtcDNA-CtDNA [21] and a representation of the average ΔCt obtained for each primer pair in three independent experiments is shown in figure 2B. Known untranscribed regions, like the plasmid origin of transfer (between P*stbA* and P*trwA*), yielded ΔCt = −8, while the most actively transcribed region corresponded to the integron cassettes, with ΔCt = 2. Overall, the transcriptional profile clearly delimitated the boundaries between transcriptional units (Figure 2.B). A comparison between promoter strengths, determined by fluorescence profiling, and mRNA abundances, measured by RT-qPCR (figure 2A, left lower panel), showed that both measurements were not linearly correlated (r^2^ = 0.49), indicating that mRNA processing and degradation also played significant roles in determining plasmid levels of expression.

**Figure 3:**
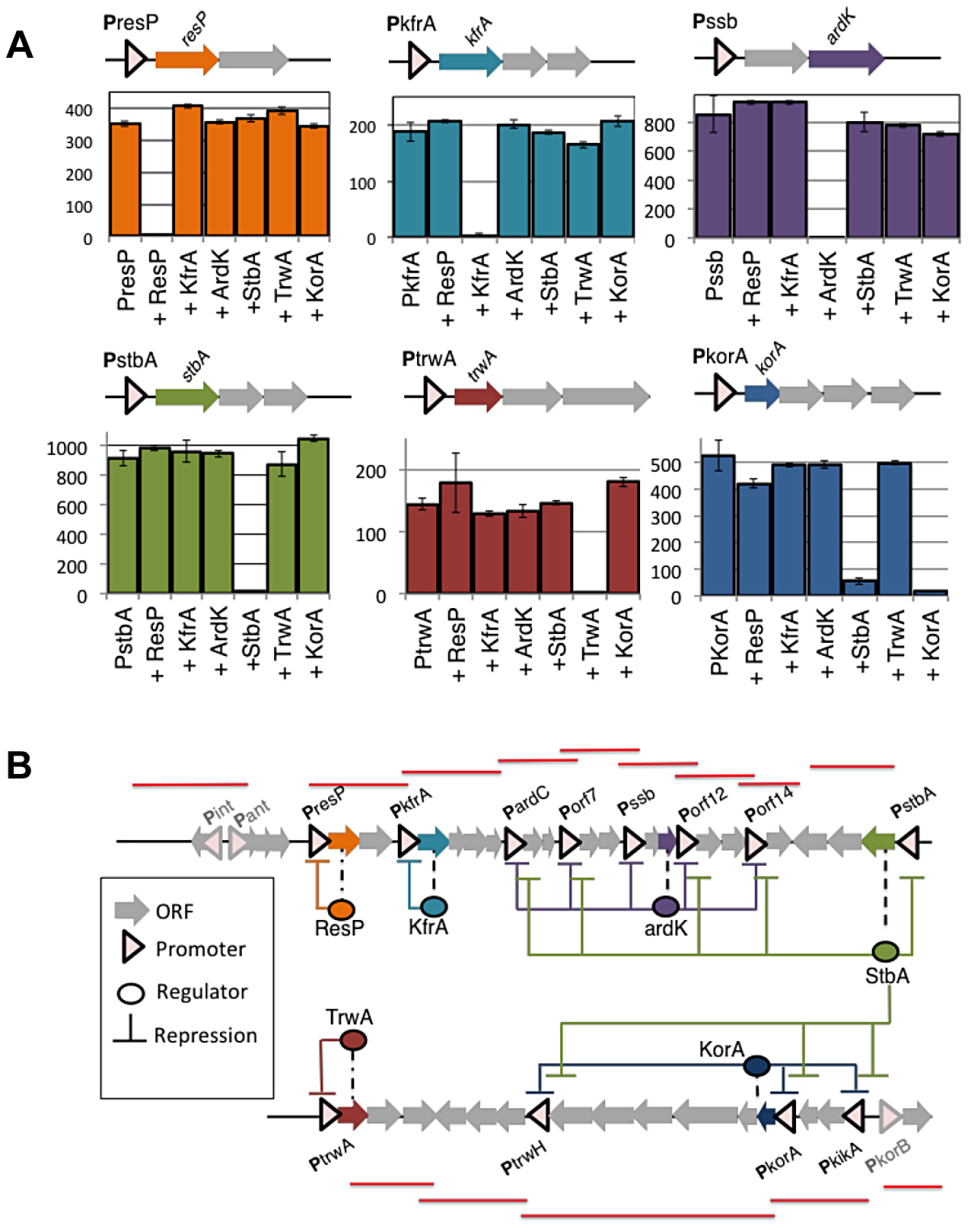
Negative feedbacks and topology of plasmid R388 transcriptional network. (**A**). Each panel shows the transcriptional activity (GFP/OD) (×10^2^) of a given promoter, either alone or in the presence of each of the six transcriptional repressors (ResP, KfrA, ArdK, StbA, TrwA and KorA). Repressors were produced from a co-residing expression vector pBAD33, and the negative control indicates the GFP/OD (×10^2^) values obtained in the presence of the empty vector. The upper diagrams show the location of each regulator with respect to its cognate promoter in plasmid R388 (**B**) A graphical representation of expression profiling data (shown in Supporting figures S3 and S4) unveils the topology of the regulatory circuitry. Coloured arrows indicate the position of transcriptional regulators within plasmid R388 genome (ResP in orange, KfrA in blue, ArdK in purple, StbA in green, TrwA in red and KorA in navy blue). The regulatory links are coloured according to the same code. Red lines shown over the ORF map correspond to the transcriptional units identified in Figure 2.

To determine the topology of the plasmid regulatory network, we tried to ascribe each plasmid promoter to its cognate regulators. ORFs from the plasmid genome that were either orphan, or showed homology to known transcriptional regulators, were considered potential candidates to encode a plasmid regulator. These ORFs were cloned in expression vector pBAD33, and the transcriptional activity of plasmid promoters was measured in the presence of all putative regulators. Expression profiles are shown in supporting figures S1 and S2, and steady-state levels are indicated in Supporting Table S1. Results allowed the identification of six plasmid proteins (ResP, KfrA, ArdK, StbA, TrwA and KorA) able to repress at least one of the plasmid promoters. (Supporting Table S1 and Supporting figure S1). Among the regulators identified, we did not find any transcriptional activator. All regulators were repressors involved in negative feedback loops (Figure 3A). Three of them controlled only their own promoter, thus constituting simple negative feedback loops (ResP, KfrA and TrwA). Another three (ArdK, StbA and KorA) controlled more complicated circuits. Protein ArdK repressed expression from **P** *ardC*, **P** *orf7*, **P** *orf12*, **P** *orf14* and **P** *ssb*, its own promoter (Supporting fig. S1). All these promoters direct the transcription of genes involved in the stable maintenance of the plasmid [3]. Therefore, ArdK seems to regulate the vegetative maintenance of plasmid R388. Similarly, protein KorA was found to regulate **P** *trwH*, **P** *korA*, **P** *kikA*, **P** *korB* and its own promoter, **P** *korA* (Supporting Fig. S2). All these promoters are responsible for expression of the Type IV secretion system, involved in plasmid conjugation. Therefore, KorA acted as the main transcriptional regulator for expression of the conjugative pilus. The third protein involved in a complex regulatory circuit was StbA. Gene *stbA* is part of an operon responsible for plasmid segregation [22] and was found to decrease **P** *stbA* transcription 50-fold (Figure 3A, Supporting Table S1). StbA also repressed transcription from promoters **P** *ardC*, **P** *orf7*, **P** *orf12* and **P** *orf14*; indicating that its target repertoire overlaps with that of ArdK (Supporting Fig. S1). Similarly, StbA repressed the promoters targeted by KorA, although the level of repression exerted was significantly lower (2 to 10-fold decrease compared to the 90-fold decrease produced by KorA on **P** *trwH*) (Supporting Fig. S2). Interestingly, previous work on StbABC showed that this operon balances plasmid partition and conjugation [23]. Results presented here indicate that StbA acts as a common regulator for genes involved in the vegetative and conjugative functions of the plasmid.

These results allowed us to determine the topology of plasmid R388 transcriptional network, which is depicted in Figure 3B. The network is completely dominated by negative repression, and promoter activation will depend on signals levering the action of plasmid repressors. In order to identify the signals that the network responded to, we challenged the plasmid with a plethora of environmental changes. We modified growth medium (LB, minimal M9), temperature (30, 37 and 42°C) and tested the presence of stressing agents, like sub-inhibitory concentrations of antibiotics and common triggers of the SOS response (Materials and Methods). As judged from fluorescent expression profiling, none of these signals was found to specifically activate any promoter in the network (Supporting figures S3, S4 and S5). The possible effect of *Escherichia coli* recipient cells was also tested by co-culture in liquid media with plasmid free cells (Supporting figure S6). Since plasmid R388 can only conjugate on solid surfaces [24], these conditions prevented horizontal transfer of the plasmid, while allowing the donors to sense any potential signal from the recipient cells. Again, the regulatory network was unresponsive (Supporting figure S6), indicating that, in the conditions tested, the network did not respond to any diffusible signal from the recipient cells. Altogether these results indicated that plasmid R388 does neither respond to pheromones (unlike many plasmids from *Gram +* bacteria [15]), nor quorum sensing signals (unlike Ti plasmids from *Agrobacterium* [16], [17]) nor SOS inducing agents (like many phages and ICEs [25])

The absence of responses against environmental challenges, DNA damage or quorum signals suggested that plasmid regulation is disconnected from the main sensory circuitry of the host cell. However, sensing is not the only function that transcriptional regulation can undertake; generating temporal programs, or guarding the cell homeostasis are also adaptive functions that arise purely from the internal dynamics of regulatory systems. In order to study the internal dynamics of the plasmid network, we used a simple quantitative model. Since all transcriptional regulators in the plasmid were self-repressors (Figure 3), we used a simple ordinary differential equations (ODE) model of a negative feedback loop. Let X denote the mRNA and Y the protein concentrations for a given feedback loop, the system of differential equations that describe the system follows:
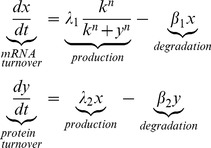
(1)


**Figure 4:**
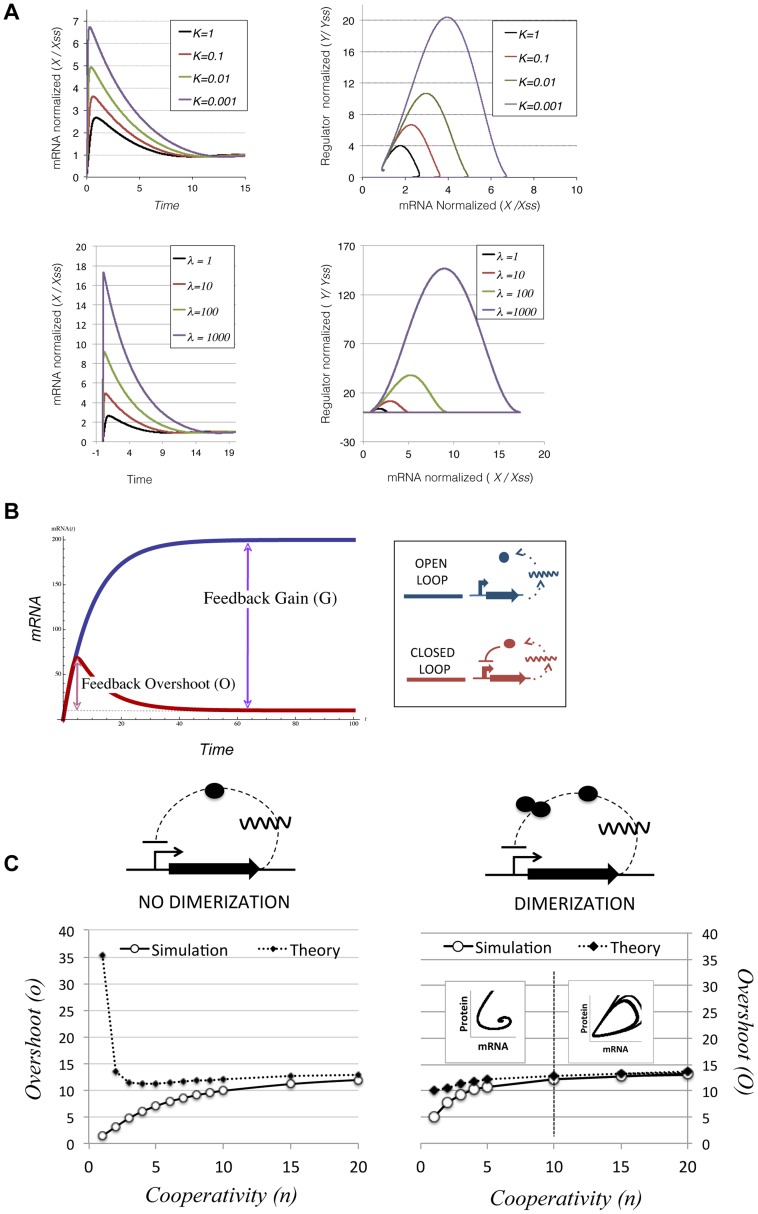
Transcriptional overshooting and its relationship with feedback gain. (**A**) Numerical simulations showing the effect of increasing feedback gain on the magnitude of the transcriptional overshoot. Left panels show the relative abundance of mRNA (X) normalized by its steady-state value (Xss) along time. Right panels show the phase-plane portrait of the system, where the x axis corresponds to the normalized mRNA values (variable X in Ec.1) and the y axis corresponds to the normalized regulator levels (variable Y in Ec.1). Values were normalized by their respective steady-state levels. Upper panels show the effect of increasing the feedback gain by decreasing the feedback constant K. Simulations were performed with λ1 = 10, β2 = 0.2, λ2 = 10, β1 = 1 and n = 1. Lower panels show the effect of increasing the feedback gain by increasing the intrinsic transcription rate λ1. Simulations were performed with λ1 = 0.01, β2 = 0.2, λ2 = 10, β2 = 1 and n = 1. The figure shows that the maximal values of X and Y grow as the feedback gain is increased, either by decreasing K or increasing λ1 (**B**) Scheme showing the theoretical time evolution of an open loop (blue) and a closed negative feedback loop (red). The feedback gain (G) corresponds to the ratio between the steady states of both systems, being all parameters equal (blue dashed line). The overshoot (O) corresponds to the transient production above the steady-state levels experienced by the closed loop when starting from initial conditions t = 0, x = 0, y = 0 (red dashed line) (**C**) Performance of the theoretical approximation described in Ec. 4 , compared to numerical simulations. Both panels show results obtained by numerical integration of Ec.1 (white dots) and predicted overshoots obtained from Ec. 4 (black dots). All simulations and calculations were done in a system with parameters k = 0.01, λ1 = 10, β2 = 0.2, λ2 = 10, β1 = 1 and changing the cooperativity of the repression (n, x axis). The left panel corresponds to a system where regulator Y is allowed to repress its own synthesis immediately after translation, while the right panel corresponds to the same system but including the requirement of Y dimerization before binding to DNA. Dimer formation is simulated by a simple ODE with Ka = 0.1 and Kd = 0.01. The inner graphs on the right chart show the phase-portrait of the system, with mRNA on the x axis and regulator concentration on the y axis. As shown in the figure, when the number of binding sites is higher than n = 10 the system becomes cyclostationary, opening the possibility of periodical bursts of transcription.

This equation is based on the assumption that, in the absence of repressor binding, mRNA is transcribed at rate λ_1_, and translated at rate λ_2._ Repressor binding is modelled following simple mass-action kinetics. This binding is characterized by a half maximal binding constant *k*, which is the ratio between the dissociation and binding constants (*k = k_off_/k_on_*). The model allows cooperative binding, with cooperativity index *n* (*n* = 1 for non cooperative binding). Parameters β_1_ and β_2_ correspond to the degradation rates of mRNA and the regulator, respectively. This simple ODE model has been extensively used in the literature, and was shown to confer different properties, such as decrease the response time and increase the stability of transcriptional sensory systems[26], [27]. These properties are characteristic of negative feedback loops whose components (mRNAs and proteins) are in steady state. However, apart from these and other steady-state properties, NFLs are known to exhibit transient behaviours while adjusting to the steady-state. In electrical engineering it is well known that NFLs can overshoot before reaching steady-state when they start from initial zero conditions (x = 0 , y = 0 at t = 0). In biological contexts, this property has received little attention, the reason being that daughter cells inherit not only the chromosome but also a proportional part of its regulatory elements. Thus, in the normal life of bacteria, transcriptional NFLs do not usually experience situations with zero concentration of its constituents. However, conjugative plasmids have a specific mechanism of invasion, entering a cell in the form of ssDNA, without accompanying mRNAs or transcriptional regulators. Simulations of Eq 1. mimicking these conditions produced an overshoot, showing that both the mRNA and the protein experienced a transitory burst and then relaxed to their steady state values (Figure 4A). While mathematical analysis indicated that a temporal lag between the mRNA and the protein was enough to produce overshooting (Supporting Text S1), computational analysis indicated that the magnitude of this overshoot is heavily dependent on the parameters of the system. Defining the magnitude of the overshoot as the ratio between the maximal levels reached by X (X_max_) and the value of X at steady state (X_ss_), simulations showed that increasing the promoter strength (λ) or decreasing K (increasing the strength of the repression, i.e. the affinity of the regulator for its cognate site) increased correspondingly the size of the transcriptional overshoot (Figure 4A). This dependency strongly suggested that there should be a correlation between the overshoot and the relative strength of the repression exerted by the NFL. The relative strength of the feedback can be expressed in terms of feedback gain (G) (Figure 4B). We define G as the ratio between the steady states shown by the open loop (without the repressor) and the closed loop (with the repressor)
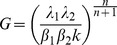
(2)


This expression indicates that the feedback gain is directly dependent on the transcriptional/translational strength (*λ_1_ λ_2_*) and inversely correlated to the feedback constant *k*. Similarly, the overshoot (O) can be expressed as the ratio between the maximum value on X divided by its steady state. Then, by linearizing X before the onset of the repression loop we can approximate O as:
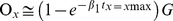
(3)


This approximation indicates that the stronger the gain (G) the higher the overshoot will be. This approximation holds for highly non-linear systems, with high values of n (Figure 4C, left panel). However, if we introduce a dimerization step where two monomers of repressor Y need to interact to form an active dimer, the approximation holds for all *n* (Figure 4C, right panel). The fact that nearly all transcription factors from Prokaryotes act as multimers indicates that this is a conservative assumption [28]. Equation 3 indicates that O is proportional to the gain G, and to the time to reach the maximal value of X (in the limit t =  = >∞, e^−βt^≈0 and O≈G). This means that O increases with higher delays, and the higher the feedback gain, the more prominent the transcriptional overshoot will be. Previous computational analysis of other feedback loops showed similar dependencies between the intensity of the overshoot and the strength of the feedback gain [29]. Therefore, simulations and theory predicted that a network architecture based on strong promoters, tightly repressed in negative feedback loops, would exhibit significant transient overshooting after HGT. For more complex circuits of the plasmid network that are under the control of two transcriptional regulators, transient overshooting is also expected (Supporting figure S7). Due to the OR logic that rules these circuits, the overshooting was dependent on the transcriptional regulator that first achieved its effective values. This, in turn, will depend of its affinity for the target promoter (*k*) and its own transcriptional/translational strength, as in simple NFLs.

**Figure 5:**
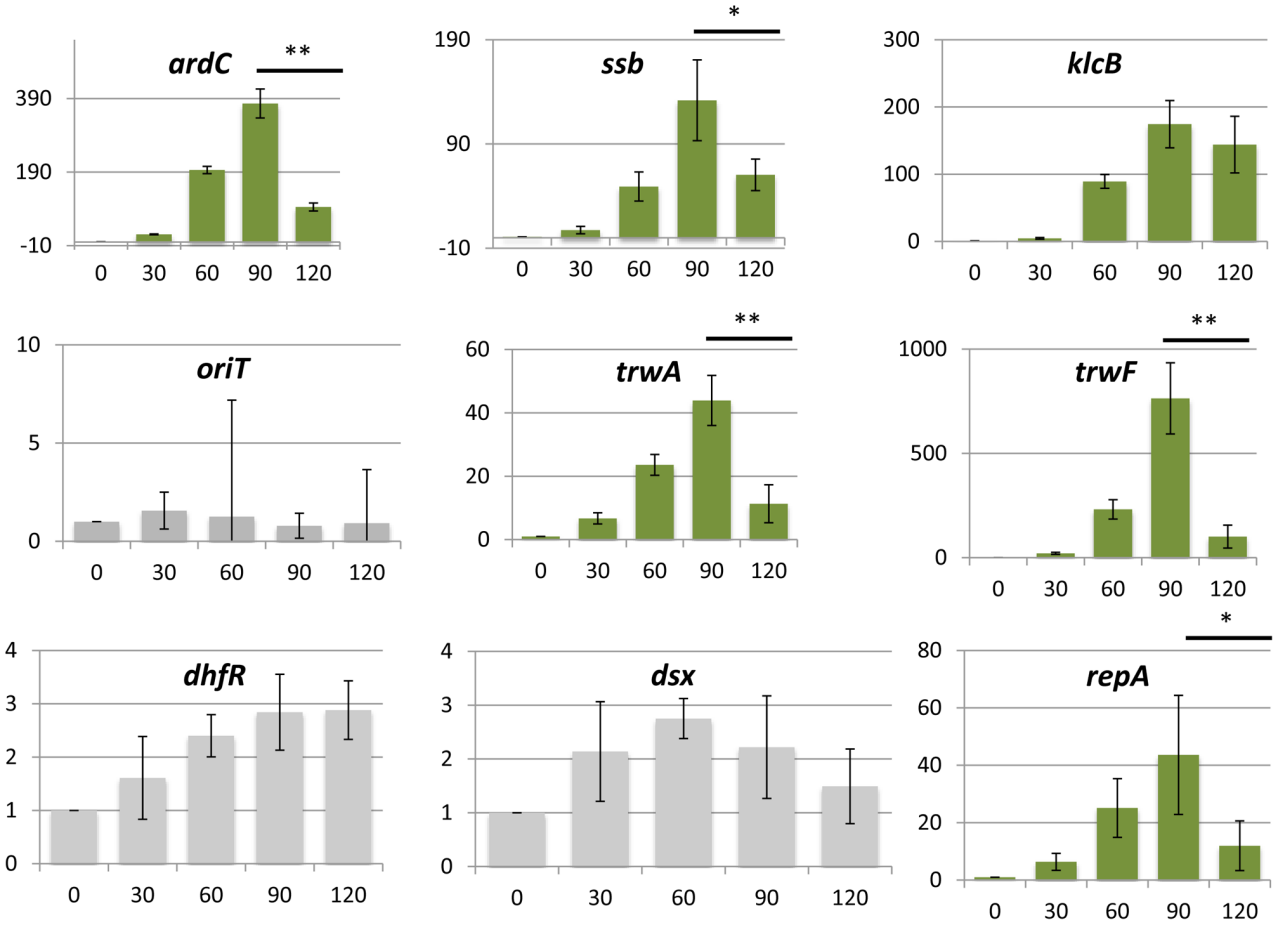
Promoter induction after horizontal transfer of the plasmid. RT-qPCR was used to measure mRNA levels. Bars indicate the relative ratio of mRNA at each time point compared to the values obtained in the absence of conjugation (time 0). Asterisks indicate the statistical significance of the differences observed * = p<0.1, ** = p<0.05 Experimental procedures and calculations are detailed in Materials and Methods and expanded results are shown in Supporting Figure S4. Measurements represent the average of three independent mRNA extractions.

To test whether this transcriptional overshoot could be detected experimentally, we determined mRNA expression levels during conjugative transfer of the plasmid. Conjugative mixes with 1∶1 donor/recipient cell ratio were allowed to mate for 0, 30, 60, 90 and 120 min. Total RNA was extracted from time samples, and expression levels from the main plasmid operons were measured by RT-qPCR. Expression levels were normalized by the results obtained from a constitutive chromosomal gene (dxs). In order to check for possible mRNA increases due to conjugative replication of the plasmid, we measured the relative increase in non-transcribed regions (*oriT*), and also in promoters that were not negatively regulated (*dhfR* gene, controlled by **P** *ant*). Experimental procedures are detailed in materials and methods. Results, shown in figure 5, indicated that the expression levels of *oriT* and *dhfR* showed limited changes, while genes controlled by negative regulation (*resP*, *ardC*, *ssb*, *klcB*, *trwA* and *trwF*) increased their relative abundance immediately after conjugation. Experimental results showed that those genes that showed the highest induction (*trwF*, *ardC*, *ssb* and *klcB*) corresponded to promoters with the higher gains (**P**trwH, **P**ardC, **P**ssb and **P**orf12). On the other hand, those promoters with lower gains (**P**trwA and **P**resP) also yielded lower overshoots (*trwA*, *repA* in fig. 5), as predicted by theory. Since conjugation is inherently asynchronous (newly formed transconjugants become donors and infect new receptors), our population measurements resolved poorly the actual kinetics of the overshoot. Also, the kinetics of the overshoot for individual NFLs will depend critically on the mRNA half-life (Eq.3), which is also likely to be variable from gene to gene. As a consequence, the decrease in the overshoot is only observable in some of the genes tested (*ardC*, *ssb*,*trwH trwA*). However, steady-state measurements (equivalent to time 0 in figure 5) indicated that all promoters would eventually return to basal levels. For the *klcB* gene, controlled by **P**orf12 promoter, which yielded no observable overshoot, it is not possible to state at this point whether the overshoot was obscured by population effects, or the parameters of this promoter did not yield any significant overshoot.

**Figure 6:**
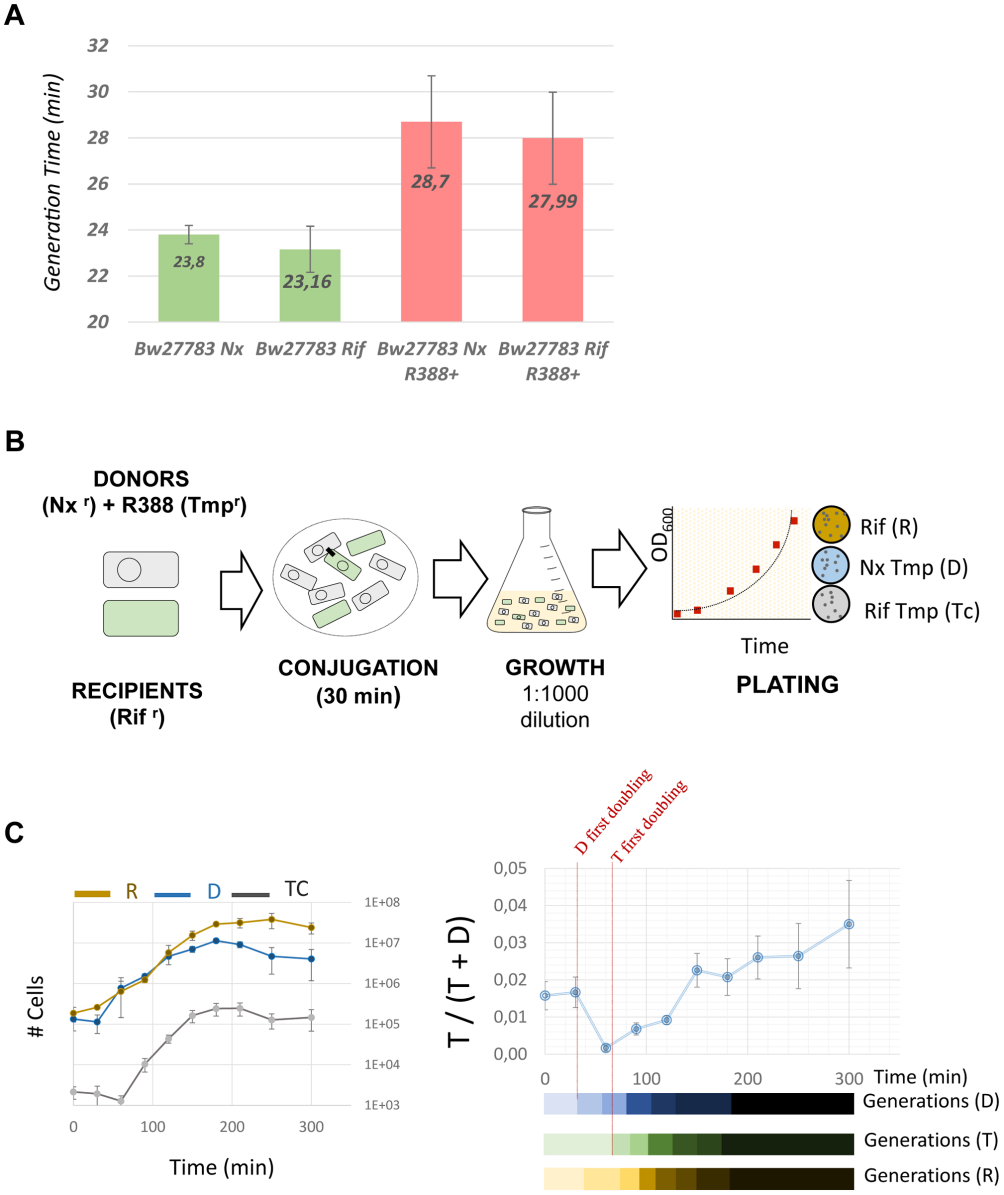
Transconjugants experience a growth deficit immediately after conjugation. (**A**) Generation times (in minutes) of *E.coli* Bw27783 with or without plasmid R388. Results represent the average and standard deviation of 12 experiments. (**B**)Scheme showing the experimental design to test the effect of plasmid conjugation in early transconjugants. Donor and recipient cells were grown in LB broth in the presence of selective antibiotics and mixed in 1∶1 conjugations on LB-Agar. Conjugation was allowed to take place for 30 minutes and cells were resuspended in fresh LB. Plate counting was used to determine de number of donors (D) recipients (R) and transconjugants (Tc). (**C**) Results of the competition experiments between D, R and Tc cells. ***(Left panel)*** Absolute numbers (cells/ml) of each species along the time course of the experiment. Each data point was measured by triplicate. ***(Right panel)*** Proportion of plasmid containing cells that are transconjugants along the time course of the experiment. Since plasmid R388 does not conjugate in liquid media, all variations in the relative proportions of Tc cells to D and R cells must be due to differences in growth. Results show a decrease in the relative abundance of Tc cells compared to D cells, that recovers after t = 90 minutes. The lower bars indicate the apparent generation times for each cell type calculated from data shown on the left panel.

This transient induction could have phenotypic effects on the host cell. Plasmids impose a burden on the host, meaning that, in the absence of positive selection for plasmid-encoded traits like antibiotic resistances, plasmids must survive as parasitic entities [30], [31], [32]. It is conceivable that a transient increase in plasmid gene expression will translate into a higher burden to the host cell. To test whether any effect on host fitness could be observed, we measured the growth rates of donor, recipient and transconjugant cells, immediately after conjugation. We used two spontaneous Rif^r^ and Nx^r^ mutants of *E.coli* strain Bw27783, which showed no observable differences in growth rate (figure 6A). Cells from both strains that had carried the plasmid for at least 10 generations exhibited a 17% increase in the generation time when compared to plasmid free cells (Figure 6A). This indicated that the plasmid exerted a measurable burden on the host Plasmid conjugation assays were performed on LB agar surfaces in a 1∶1 donor/recipient ratio, and cells were allowed to mate for 30 min. Conjugation was stopped by resuspending cells from the solid surface, cells were diluted to OD_600_≈0.01 in fresh LB, placed in agitation at 37 C and allowed to grow for 5 h (Figure 6B). Growth rates were measured by plating on selective antibiotics (materials and methods). Plasmid R388 does not conjugate in liquid media, thus any variation in the proportion of donors, recipients and transconjugants must be due to relative differences in growth rates. Results, shown in figure 6C, indicated that transconjugant cells suffered a remarkable decrease in growth rate immediately after conjugation, showing a first generation time of about 2.5× times that of donor cells. However, after this long first generation, transconjugant cells recovered, achieving the same number of divisions as donor cells for the total duration of the experiment (7 generations). Similar results were obtained when donor and recipient strains were reversed (Supporting Figure S8). The observed growth deficit in the transconjugants could be a by-product of the conjugation mechanism, which requires the piercing of the recipient cell by the transfer apparatus. To test whether this was the case, we carried out a similar experiment with a mobilizable plasmid. In this case, a small cloning vector carrying just the origin of transfer (*oriT*) of plasmid R388 was mobilized into recipient cells by means of an *oriT* ^−^ mutant of plasmid R388. Under these conditions, plasmid R388 does not move itself, but is still able to produce a conjugative pilus and thus mobilize the small vector into the recipient cells. Results show that vector mobilization did not produce a significant decrease in the growth rate of transconjugant cells (Supporting Figure S8). This indicated that the transitory deficit in growth rate was not due to cell injuries produced by the mechanism of conjugation, but was a consequence of the entry of the conjugative plasmid inside the recipient cell.

## Discussion

The intensity of HGT in the microbial world, and the prevalence of plasmids in nature indicate that plasmids are successful in colonizing microbial populations. Yet multilevel selection imposes opposing demands on plasmid physiology that require a delicate equilibrium between the expression of plasmid functions and the burden imposed on the host cell [9]. Understanding the regulatory mechanisms of plasmid transcriptional control might shed light on the way plasmids conciliate these requirements.

In this work we describe the topology and dynamics of the transcriptional network of plasmid R388, the smallest BHR plasmid from *Proteobacteria*. The network consisted exclusively of transcriptional repressors. This preference for transcriptional repression is in contrast with the situation described for the regulatory networks of bacterial chromosomes. For example, in *E.coli* the number of transcriptional activators roughly equals the number of repressors [33]. However, other transcriptional networks from BHR plasmids, like plasmid RP4, were also found to depend solely on transcriptional repressors [11]. In plasmid R388, transcriptional repression was exerted mainly in the form of negative feedback loops. These feedback loops showed high gains (defined as the ratio between the expression levels of the open and the closed feedback loops). Although we are not aware of any systematic, quantitative study of a plasmid regulatory network, several independent studies have reported that the regulatory network of plasmid RP4 contains strong promoters that are kept tightly repressed by the plasmid regulators [14], [34], [35],[36],[37],[38]. Remarkably, plasmids R388 and RP4 show similar broad host ranges, but they are not phylogenetically related [3], [4], [39]. This indicates that both plasmids, which presumably suffer from analogous selective constrains, have independently evolved transcriptional networks with analogous topologies.

Simulations and theory indicate that whenever a negative feedback loop has a gain higher than 1 and a certain time delay between the mRNA and the regulatory protein, the system would show transient overshooting (Eq 3 and figure 4A). The actual production of the overshoot requires the system to begin with zero initial concentration of transcriptional repressors (t = 0, x = 0, y = 0, in Eq 1), allowing the separation of timescales to produce a period of repressor-free transcription. For conjugative plasmids, this situation is met every time the plasmid enters into a new cell by conjugation. In fact, any negative feedback loop that undergoes conjugation is likely to experience transient overshooting. It has been known for a long time that a lysogenic phage transferred by *Hfr* conjugation (an artificial system that allows the horizontal transfer of the entire chromosome), can become lytic when entering into a new host [40]. A similar behavior was also observed when an RFP-TetR autogenously regulated cassette was inserted in the *E.coli* chromosome and transferred by *Hfr* conjugation into a new cell [41]. Transient overshooting is therefore an epiphenomenon associated to negative feedback loops that experience some sort of “genome rebooting”, a condition where the transcriptional/translational machinery is present, but the regulatory network is transitory absent.

Simulations and theory also indicated that the overshoot is expected to be higher whenever the feedback loop has a high gain. Plasmid promoters were shown to contain feedback loops with characteristic high gains. RT-qPCR analysis showed a transcriptional burst in 5 out of 6 plasmid promoters subjected to NFLs, when the plasmid transferred horizontally into a new population (Figure 5). Untranscribed regions (*oriT*), or plasmid genes that were not under the control of a negative regulator (*dhfR*), did not show similar increases (Figure 5). This indicates that the observed effect is not due to conjugation increasing the abundance of plasmid molecules within the population. Moreover, given that the conjugative mix contained a 50% ratio of donor/recipient cells, the maximal increase that conjugation could cause is 2-fold. The increase of mRNA abundance was not due to cell growth either, since results were normalized by the increase experienced by a constitutively expressed chromosomal gene (*dxs*). Gene *dxs* showed a maximal increase of 2-fold, indicating that cell growth is a minor contributor to the observed bursts in mRNA levels. These results cannot be considered absolute quantifications of the transcriptional overshooting, because our measurements involved entire populations (which contained donor and recipient cells), and bacterial conjugation is an asynchronous process. However, although our quantitative results might be blurred by population effects, the general trend predicted by theory and simulations was sound: those promoted that showed higher gains also showed the higher overshoots.

Plasmids are known to produce fitness deficits on their hosts. This effect has been usually ascribed to the metabolic burden imposed by expression of plasmid genes. Therefore, any increase in expression levels caused by transient overshooting might have its counterpart in the growth rate of the host cell. We measured the growth rates of newly formed transconjugants and found that the plasmid induced a deficit that was transitorily high (250% increase in generation time), relaxing later to a 17% increase compared to plasmid free cells. This was not caused by any physical damage produced by the mechanism of conjugation, and correlates in time with the induction of plasmid genes after transfer. Altogether, these results strongly suggest that overshooting after HGT has a measurable impact on the host growth rate. Although this kind of effect has been traditionally ascribed to metabolic burden, it is also possible that the toxic effects of specific plasmid proteins could contribute. Since the growth deficit roughly corresponds to the time of overshoot decay (figure 5 and figure 6C) the most plausible explanation is that growth deficit be caused by the transcription/translation of the plasmid genes. This would also explain why, when the recipient cells recover, they grow as fast as recipient cells for a few generations.

One intriguing question then is why has the plasmid evolved a network based exclusively on NFLs, when this motif is likely to overshoot after conjugative transfer, temporarily hampering the host growth rate? Other broad host range plasmids show convergent architectures, suggesting that despite this temporary fitness deficit, negative feedback might have some adaptive property for the plasmid lifestyle. Indeed, negative feedback has been shown to exhibit a number of adaptive properties, speeding up the response time of sensory regulatory networks [27], reducing transcriptional noise [42], [43], driving noise to higher frequencies and allowing easier filtering [44]. Speeding up the response is a property associated to sensory systems, and so far the plasmid network has not shown responses to any specific signals. Noise control might be more interesting for plasmids, given that plasmid replication is extremely sensitive to fluctuations [45], [46]. However this problem is restricted mainly to replication, and does not explain why the same regulatory strategy is widespread in the entire plasmid backbone.

It is also possible that transient overshooting provides an adaptive benefit for the particular lifestyle of conjugative plasmids. Plasmids spread horizontally, by invading new cells, and vertically, as the host cell reproduces. Like many other parasites that share this double reproductive strategy, plasmids suffer from opposing selective forces, summarized in the observation that increased infectivity usually results in increased virulence. This inverse relationship is well known in plasmids and phages [30], [31], [32], and if a given plasmid increases the expression levels of its own plasmid products (especially those that are *cis*-acting), it would also increase its intracellular fitness, at the cost of penalizing the host [9]. Penalizing the host, in turn, decreases the ability of the host cell to compete with its neighbours [9], and thus the plasmid experiences lower vertical transmission rates. Although both selection processes are intrinsically in conflict, the timescales involved in each of them are different. The decrease on host fitness imposed by the plasmid metabolic burden is usually low (% in the case of plasmid R388), meaning that intercellular selection acts by the accumulation of small fitness deficits over long periods of time [5], [6], [8]. On the other hand, intracellular selection is more pronounced in the initial stages of infection, since a cell that has received the plasmid is still susceptible to superinfection until the surface exclusion systems have been deployed [9] [47]. Therefore, it is in the interest of the first plasmid that enters into a cell to block the entrance of other plasmid copies, and to reach the steady-state copy number as soon as possible. Transcriptional overshooting after HGT would allow the plasmid to produce a vigorous transcriptional response when intracellular selection is more acute. The transient nature of this response would guarantee that the long-term effects on intercellular selection are minimized. Indeed competition experiments showed that, despite the severe initial effect on the host growth rate, transconjugants recovered quickly and were able to achieve the same number of cell divisions as the original donors. Note also that since transconjugants are able to act as donors, conjugation results in an infectious process that proceeds geometrically in the population. If overexpression of conjugative functions results in increased transfer efficiency, a transient overshoot would provide the invading plasmids with higher infectivity. This property will be maintained as long as new cells are infected. If the availability of possible receptors decreases, the overshoot transient nature guarantees that the plasmid population relaxes to a “silent” state, minimizing the burden on the host and improving vertical transmission. Such a mechanism would provide the plasmid population with a mechanism to switch from horizontal to vertical reproduction modes depending on the availability of susceptible receptors. Other lines of evidence also point to this possibility. The *stbABC* operon of plasmid R388 has been shown to balance the requirements for vegetative stability and conjugative transfer [23]

The fact that transient overshooting is linked to genome rebooting is also interesting from a synthetic biology perspective. Plasmids are nature counterparts of genomic transplantations. In fact, they can be considered as genetic devices for the unidirectional injection of genomes into suitable recipient cells. So far, efforts to transplant whole chromosomes have been restricted to species that share a high degree of genomic identity [48]. A close phylogenetic relationship implies that the regulatory networks of both species might show some cross-reactivity, which could be necessary to control the transplanted chromosome until it has built up its own regulatory system. Distantly related species, however, might show no cross-reactivity between their regulatory networks. Broad host conjugative plasmids are able to invade a wide variety of distantly related species. If we want to expand the range of possible transplants, we need to deal with problems identical to those faced by conjugative plasmids. In particular, how can a genome start up from just DNA and the transcriptional/translational machinery? Negative regulation, with high feedback gains and transient overshooting might be the solution evolved by natural plasmids.

## Materials and Methods

### Promoter library construction

Strains used were *Escherichia coli* C41 (*ompT hsdS*_B_ (r_B_^−^ m_B_^−^) *gal dcm* (DE3)), *E. coli* Bw27783 (*lacI* ^q^ *rrnB3* Δ*lacZ4787 hsdR514* Δ(*araBAD*)*567* Δ(*rhaBAD*)*568* Δ(*araFGH*) Φ(Δ*araEp* P_CP8_-*araE*)) [49] and *E. coli* JM109 (*recA1, endA1, gyrA96, thi, hsdR17, supE44, relA1, Δ(lac-proAB)/F′ [traD36, proAB^+^, lacI^q^, lacZΔM15]*) Primer oligonucleotides (Supporting table S2) were designed to flank each R388 intergenic region longer than 30 bp, according to R388 genomic sequence (Genbank accession number BR000038) and purchased from Sigma. R388 plasmid DNA was extracted using Sigma GenElute Miniprep kit and used as template for PCR amplification. PCR amplification was carried out with Vent DNA polymerase (Biolabs) and consisted of 95°C for 10 min, then 28 cycles of 95°C for 30 s, 55°C for 30 s, 72°C for 30 s and a final step of 72°C for 5 min. PCR products were digested with *XhoI* and *BamHI* at 37°C for 2 h and the products purified using QIAquick Gel Extraction Kit (Qiagen). Digested and purified fragments were ligated into pUA66 plasmid DNA using T4 ligase (Roche) with overnight incubation at 16°C. Transformation was accomplished by electroporation into Bw27783 strain. Transformants were selected in LB-agar plates containing 25 µg/ml kanamycin. Positive colonies were detected by PCR using pZE0.5 and pZE0.6 primers. DNA from positive colonies was extracted and insertions sequenced using the same primers as above. The set of reporter plasmids obtained is indicated in Supporting table S2.

Plasmid R388 was transferred to the reporter strains by conjugation. Donor bacteria were *E. coli* JM109 containing plasmid R388 and recipient bacteria were *E. coli* BW27783 containing the corresponding reporter plasmid. Conjugations were carried out as previously described [50].

### Fluorescent expression profiling

Transcriptional activity was determined by GFP expression profiling as described in [51]. Reporter strains were inoculated into enriched M9 Medium (M9 + casaminoacids 0.2%+ glycerol 0.5%) to which kanamycin (25 µg/ml) was added. To test the effect of subinhibitory concentrations of antibiotics, we used rifampicin and chloramphenicol following the protocol described in [52]. Results represent the average of at least 4 independent measurements. The S.O.S. response was induced exposing the cells to 5 or 10 seconds of UV light (254 nm, 15W). Mitomycin C was used at a final concentration of 5 µg/ml.

### Effects of regulatory proteins

Selected R388 ORFs containing potential transcriptional regulators were PCR amplified with primers indicated in table S1. The resulting DNA segments were cloned in plasmid pET3a (Addgene). The genetic manipulation techniques were the same as those described above, except that *NdeI* and *BamHI* restriction endonucleases were used for cloning PCR products in pET3a expression vector. After transforming to *Escherichia coli* C41 strain, protein expression was induced by adding 0.1 mM IPTG to exponentially growing cultures and visualized by denaturing polyacrylamide gel electrophoresis (data not shown). Then each gene was subcloned in plasmid pBAD33 using *XbaI*-*Hind* III endonucleases. Plasmids obtained (table S2) were transformed to *E. coli* Bw27783 containing the corresponding reporter plasmids for further analysis. To determine the effect of potential regulatory proteins, pAR expression plasmids (Supporting table S2) were transformed into *E. coli* Bw27783 containing the corresponding reporter plasmid. Protein expression was induced by adding appropriate concentrations of arabinose [51] to M9-broth and fluorescence per OD unit (GFP/OD) was determined and compared to that produced by the same reporter strain when containing the empty expression vector pBAD33.

### Quantification of mRNA levels

Total RNA was extracted from E.coli Bw27783 containing plasmid R388 and grown in LB media at 37C. Cells were harvested at OD600 = 0.5 and RNA was extracted using RNAEasy kit (Quiagen). Total RNA concentration was quantified using Experion RNA StdSens Analysis kit (Biorad). cDNA was obtained by reverse transcriptase (Omniscript, Qiagen) and the relative concentration of the target genes was determined by qPCR (ICycler, Biorad) using the IQ SYBR Green Supermix kit (Biorad). To determine the cDNA abundance, the threshold cycle (Ct) was determined using the ICycler software. A total of 66 primer pairs were manually designed to cover the entire genome of the plasmid, and the efficiency of each primer pair was determined measuring the Ct obtained from 3 different DNA concentrations (2.5, 5 and 10 ng). The sequence and efficiencies of each primer is shown in Supporting table S2. cDNA reactions were performed from 300 ng total RNA concentration and results were normalized by the Ct obtained from 5 ng of plasmid DNA purified by alkaline lysis.

### RT-qPCR measurements of gene expression during conjugation

To determine the relative expression of plasmid genes during conjugation, 1 ml samples of donor (*E.coli* Bw27783 +R388 plasmid) and recipient (*E.coli* Bw27783) cultures were mixed in a 1∶1 ratio, pelleted and resuspended in 100 l of fresh LB. Cells were then spread on LB-Agar and incubated at 37C for 30, 60 or 90 min. After each conjugation period, cells were resuspended in 2 ml of fresh LB and total RNA was extracted as described in the previous paragraph. Primer pairs used are shown in supporting table. S2 For each independent experiment measurements were performed in duplicate, and a total of 4 independent experiments were performed for each time point. In order to avoid artefacts introduced by cell manipulation, the RNA concentration at time 0 was determined following the same manipulation procedure, but cells were immediately resuspended after being plated in LB-Agar. The relative concentration of target RNAs (r) was determined by r = E^(ΔCt)^ where E is the efficiency of the primer pair, calculated as in [21], and ΔCt = Ct_T = t_−Ct_T = 0_. Results were normalized to the increase experienced by a chromosomal gene (*dxs*) using the ΔCt method, where ΔCt = (Ct_T = t_−Ct_T = 0_)_probe_ [21]. Relative error was propagated using the standard propagated error formula. For the relative amount of a test mRNA (Ct) with respect to a given standard (Ctdxs) the aforementioned formula yields σ_x_/<x> = ln(2) (*var*(Ct)+*var*(Ct_dxs_)−2*cov*(Ct,Ct_dxs_))^−2^ , where *var* stands for the variance and *cov* for the covariance. The statistical significance was ascertained using a one handed *t* test.

### Competition after conjugation experiments

Two spontaneous mutants resistant to rifampicin and nalidixic acid from *E.coli* Bw27783 were obtained by plating in selective antibiotics. The stability of the mutation was tested and the strains were used as donor/recipients in conjugation experiments. Growth rates were determined from cells growing in LB broth at 37 C with agitation, and results showed that both strains had indistinguishable division times in such conditions (n = 12). In order to measure the growth rate of donor, recipient transconjugant cells, we performed conjugations for t = 30 minutes. Saturated cultures, grown overnight in LB at 37C, were and diluted 1/1000 in fresh LB until cells reached an OD600 of 0.1. Donor and recipient cells were mixed in a 1∶1 ratio and concentrated 1000 times. A total of 15 microliters were deposited onto a LB agar plate and let at 37C for 30 minutes to allow plasmid transfer. Cells were then resuspended in 3 ml of LB broth and allowed to grow at 37C with agitation. Time samples were obtained every 30 min, and cells were diluted in 1×PBS to count the number of donor, recipients and transconjugants by plating in selective antibiotics. Plating of early time points was performed 30 minutes after PBS resuspension, to allow the antibiotic markers to express to adequate levels. We checked that this treatment did not artificially increased the number of cells due to growth in the PBS dilution. We plated dilutions from 10^−1^ to 10^−6^. The error introduced by the dilution was measured obtaining values typically around cv = 0.1–0.2. This error was propagated to the actual number of cells and accounts for most of the variability observed in the results.

### Computational analysis

Numerical integration of the ODE system was performed in Matlab (Mathworks) using the ODE23 algorithm. ODE23 is a Runge-Kutta algorithm with automatic step size. The absolute and relative tolerances were set at 10^−10^ , tspan = 1000.

## Supporting Information

Figure S1 Expression profiles of the replication and maintenance promoters in the presence or absence of their transcriptional regulators. Each panel shows the expression profile of the reporter plasmid indicated above the panel (cloned promoter indicated in brackets). Expression profiles correspond to promoter alone (black lines), in the presence of plasmid R388 (red lines), or when different regulators are expressed from a co residing pBAD33 expression vector (blue and green lines). The effect of the transcriptional regulators was tested without arabinose (darker lines, ara −) and with maximum arabinose induction (lighter lines, ara +). Some transcriptional regulators were found to decrease the growth rate when induced above a certain threshold. To discard effects produced by impaired growth rate we measured, for each regulator, the rank of arabinose concentration that did not impair bacterial growth (data not shown). Therefore maximum arabinose induction stands for the maximum concentration that did not produce a measurable effect on growth rate, and it is variable for each regulator (ranging from 10^−3^ to 10^−4^% (w/v)). **A**) Expression profiles from **P** *resP* and **P** *kfrA* promoters and response to ResP and KfrA respectively. **B**) Expression profiles from **P** *ardC*, **P** *orf7*, **P** *ssb*, **P** *orf12*, **P** *orf14* and **P** *stbA* promoters and response to ArdK and StbA. Data shown represents the average of at least four independent experiments.
(DOCX)

Figure S2 Expression profiles of conjugation region and response to their transcriptional regulators. Panels show the expression profiles (obtained as in Materials and Methods) from cultures containing the reporter plasmids indicated above each panel (corresponding promoter indicated in brackets). Profiles obtained with the reporter plasmid alone are indicated by black lines and by red lines when plasmid R388 was also present. Green and blue lines indicate profiles obtained in the presence of a given regulator expressed from a co residing pBAD33 expression vector. The effect of the regulators was determined both with arabinose induction (lighter lines, ara+) and without (darker lines, ara−). **A**) Expression profiles of **P**trwA containing reporter vector and response to R388 (red line) and TrwA (blue lines). **B**) Expression profiles from reporter plasmids containing **P**trwH, **P**korA, **P**kikA and **P**korB and response to KorA and StbA transcriptional regulators. Black lines represent expression profiles obtained from cultures containing the corresponding reporter vectors (indicated above each panel) and red lines indicate the profiles of the same reporter vector in the presence of a co residing R388. Green lines show the profile obtained when expression vector pAR12 (pBAD33::stbA) was present with (light green, ara+) and without arabinose induction (dark green, ara−). Blue lines indicate the expression profiles obtained with a co residing pAR13 vector (pBAD33::korA). Although **P**korB fluorescence levels decreased in response to KorA the profile is not shown since the difference was not statistically significant. C) Expression profiles of cultures containing **P**int and **P**ant reporter vectors alone (black lines) and in the presence of R388 (red lines). Data shown represents the average of at least four independent experiments.
(DOCX)

Figure S3 Effects of sub-inhibitory concentrations of rifampicin on plasmid promoters. (**A**) Expression profiles of plasmid R388 promoters, measured as described in Materials and Methods, in the presence of rifampicin 3 µg/ml. Rifampicin produced a general decrease in GFP/OD levels, either in the presence or the absence of plasmid R388. (**B**) Effect of rifampicin 3 µg/ml on bacterial growth rate. Growth curves were determined measuring OD600 at different time points. The upper panel shows the complete growth curve in a linear scale. The lower panel shows the exponential growth phase in a semi-logarithmic scale. As shown by the figure, rifampicin 3 µg/ml produced no detectable effect on the growth rate, while the presence of plasmid R388 decreased it significantly.
(DOCX)

Figure S4 Effects of SOS response on plasmid promoters. Charts show the GFP/OD values achieved in steady-state by the promoters indicated in the figure. Expression profiling was performed as described in Materials and Methods. Cells were treated with uv irradiation (254 nm, 15W) for 5 or 10 seconds. Mitomycin C was used at a concentration of 5 µg/ml. Those promoters that were induced by SOS response were marked with an asterisk (*). Pint showed a clear response to S.O.S induction either by Mitomycin C or by UV irradiation. **P**trwA showed a discrete 5 fold increase when the promoter was assayed alone, but that response could not be reproduced with co-residing plasmid R388.
(DOCX)

Figure S5 Temperature effects on plasmid promoters. Charts show the GFP/OD values achieved in steady-state by the promoters indicated in the figure. Expression profiling was performed as described in Materials and Methods. Cells were grown at 37 C overnight, then diluted 1∶10000 in fresh media, and then grown at the indicated temperatures.
(DOCX)

Figure S6 Presence of potential receptors. Expression profiles of plasmid R388 promoters, obtained as described in Materials and Methods. The effect of potential recipients for horizontal transfer was tested by co-culture with empty E.coli Bw27783. Cells were mixed at 1∶1 ratio before the measurement started. To obtain the same amount of GFP-producing cells, the volume of recipient-containing cultures was doubled. The only effect observed was a general decrease in fluorescence signal in those cultures that contained recipients. Cell quenching probably caused this unspecific effect.
(DOCX)

Figure S7 Transient overshooting in StbA/KorA Incoherent Feed Forward Loop (IFFL). In order to test whether the transient overshooting would also happen in more complex architectures apart from simple NFLs, we simulated the behavior of the KorA-StbA IFFL loop present in the conjugation region (Upper panel). The parameters were introduced according to the results depicted in Table S1, which indicate the order of promoter strengths (PstbA>PtrwH>PkorA) and indicated also the relative strengths of repression exerted by the two regulators (K_StbA_PkorA_ >>K_KorA_PkorA_ and K_StbA_PtrwH_>>K_KorA_PtrwH_). Results shown in the lower panel indicate that this IFFL architecture will also exhibit a transient overshoot.
(DOCX)

Figure S8 Growth rate deficit after horizontal gene transfer A. Growth rate after conjugation. **(Left panel)** Growth rate of Recipients (R), Donors (D) and Transconjugants (T). Cells were mixed at a 1∶1 ratio and allowed to conjugate for 30 min. at 37 C, on LB agar plates. Donors were E.coli Bw27783 Rif^r^ containing plasmid R388, and recipients were E.coli Bw27783 Nx^r^. Cells were then resuspended in liquid LB and allowed to grow. Cell numbers were obtained by plating on appropriate antibiotic combinations, as indicated in materials and methods. **(Right panel)** Proportion of plasmid-containing cells that are transconjugants along time. The x axis indicates the timespan since cells were taken out from conjugation mixtures. The y axis indicates the proportion of transconjugants over plasmid-. containing cells (donors + transconjugants). Plasmid R388 does not conjugate in liquid, thus any change in this proportion was due to growth differences. Lower bars indicate the apparent generation times for each species, calculated from the data shown in the left panel. **B.** Growth rate after mobilization. Growth rate of Recipients (R), Donors (D) and Transconjugants (T). Cells were mixed at a 1∶1 ratio and allowed to conjugate for 30 min at 37 C, on LB agar plates. Donors were *E.coli* Bw27783 Rif^r^ containing plasmid R388Δ*nic*, and the mobilizable vector pSU4910 (Cm^r^). R388Δ*nic* encodes for the entire transfer system, but lacks the *nic* site needed in cis for a DNA to be transferred by conjugation. Thus this strain is able to mobilize pSU4910 without transferring plasmid R388. Recipients were *E.coli* Bw27783 Nx^r^. Experiments were performed as in conjugation assays.
(DOCX)

Table S1 Promoter activities in the presence of plasmid R388 and plasmid transcriptional regulators.
(DOCX)

Table S2 Oligonucleotides and plasmids used in this study.
(XLSX)

Text S1 Calculations on the Gain-Overshoot relationship.
(PDF)
