# Supplementary material for: Negative Feedback and Transcriptional Overshooting in a Regulatory Network for Horizontal Gene Transfer
Source: PLoS Genet. 2014 Feb 27;10(2):e1004171. doi: 10.1371/journal.pgen.1004171 (PMC3937220; doi:10.1371/journal.pgen.1004171)
Supplement: Figure S1 — Expression profiles of the replication and maintenance promoters in the presence or absence of their transcriptional regulators. Each panel shows the expression profile of the reporter plasmid indicated above the panel (cloned promoter indicated in brackets). Expression profiles correspond to promoter alone (black lines), in the presence of plasmid R388 (red lines), or when different regulators are expressed from a co residing pBAD33 expression vector (blue and green lines). The effect of the transcriptional regulators was tested without arabinose (darker lines, ara −) and with maximum arabinose induction (lighter lines, ara +). Some transcriptional regulators were found to decrease the growth rate when induced above a certain threshold. To discard effects produced by impaired growth rate we measured, for each regulator, the rank of arabinose concentration that did not impair bacterial growth (data not shown). Therefore maximum arabinose induction stands for the maximum concentration that did not produce a measurable effect on growth rate, and it is variable for each regulator (ranging from 10−3 to 10−4% (w/v)). A) Expression profiles from P resP and P kfrA promoters and response to ResP and KfrA respectively. B) Expression profiles from P ardC, P orf7, P ssb, P orf12, P orf14 and P stbA promoters and response to ArdK and StbA. Data shown represents the average of at least four independent experiments. (DOCX) [file pgen.1004171.s001.docx]

**Supporting Figure 1. Expression profiles from replication & maintenance promoters**
