# Supplementary material for: Negative Feedback and Transcriptional Overshooting in a Regulatory Network for Horizontal Gene Transfer
Source: PLoS Genet. 2014 Feb 27;10(2):e1004171. doi: 10.1371/journal.pgen.1004171 (PMC3937220; doi:10.1371/journal.pgen.1004171)
Supplement: Figure S2 — Expression profiles of conjugation region and response to their transcriptional regulators. Panels show the expression profiles (obtained as in Materials and Methods) from cultures containing the reporter plasmids indicated above each panel (corresponding promoter indicated in brackets). Profiles obtained with the reporter plasmid alone are indicated by black lines and by red lines when plasmid R388 was also present. Green and blue lines indicate profiles obtained in the presence of a given regulator expressed from a co residing pBAD33 expression vector. The effect of the regulators was determined both with arabinose induction (lighter lines, ara+) and without (darker lines, ara−). A) Expression profiles of PtrwA containing reporter vector and response to R388 (red line) and TrwA (blue lines). B) Expression profiles from reporter plasmids containing PtrwH, PkorA, PkikA and PkorB and response to KorA and StbA transcriptional regulators. Black lines represent expression profiles obtained from cultures containing the corresponding reporter vectors (indicated above each panel) and red lines indicate the profiles of the same reporter vector in the presence of a co residing R388. Green lines show the profile obtained when expression vector pAR12 (pBAD33::stbA) was present with (light green, ara+) and without arabinose induction (dark green, ara−). Blue lines indicate the expression profiles obtained with a co residing pAR13 vector (pBAD33::korA). Although PkorB fluorescence levels decreased in response to KorA the profile is not shown since the difference was not statistically significant. C) Expression profiles of cultures containing Pint and Pant reporter vectors alone (black lines) and in the presence of R388 (red lines). Data shown represents the average of at least four independent experiments. (DOCX) [file pgen.1004171.s002.docx]

**Supporting Figure 2. Expression profiles from transfer promoters**
