# Supplementary material for: Negative Feedback and Transcriptional Overshooting in a Regulatory Network for Horizontal Gene Transfer
Source: PLoS Genet. 2014 Feb 27;10(2):e1004171. doi: 10.1371/journal.pgen.1004171 (PMC3937220; doi:10.1371/journal.pgen.1004171)
Supplement: Figure S3 — Effects of sub-inhibitory concentrations of rifampicin on plasmid promoters. (A) Expression profiles of plasmid R388 promoters, measured as described in Materials and Methods, in the presence of rifampicin 3 µg/ml. Rifampicin produced a general decrease in GFP/OD levels, either in the presence or the absence of plasmid R388. (B) Effect of rifampicin 3 µg/ml on bacterial growth rate. Growth curves were determined measuring OD600 at different time points. The upper panel shows the complete growth curve in a linear scale. The lower panel shows the exponential growth phase in a semi-logarithmic scale. As shown by the figure, rifampicin 3 µg/ml produced no detectable effect on the growth rate, while the presence of plasmid R388 decreased it significantly. (DOCX) [file pgen.1004171.s003.docx]

**Supporting Figure S3 Effect of sub-inhibitory concentrations of Rifampicin on plasmid promoters**
