# Supplementary material for: Negative Feedback and Transcriptional Overshooting in a Regulatory Network for Horizontal Gene Transfer
Source: PLoS Genet. 2014 Feb 27;10(2):e1004171. doi: 10.1371/journal.pgen.1004171 (PMC3937220; doi:10.1371/journal.pgen.1004171)
Supplement: Figure S4 — Effects of SOS response on plasmid promoters. Charts show the GFP/OD values achieved in steady-state by the promoters indicated in the figure. Expression profiling was performed as described in Materials and Methods. Cells were treated with uv irradiation (254 nm, 15W) for 5 or 10 seconds. Mitomycin C was used at a concentration of 5 µg/ml. Those promoters that were induced by SOS response were marked with an asterisk (*). Pint showed a clear response to S.O.S induction either by Mitomycin C or by UV irradiation. PtrwA showed a discrete 5 fold increase when the promoter was assayed alone, but that response could not be reproduced with co-residing plasmid R388. (DOCX) [file pgen.1004171.s004.docx]

**Supporting Figure S4. Effects of the SOS response on plasmid promoters.**
