# Supplementary material for: Negative Feedback and Transcriptional Overshooting in a Regulatory Network for Horizontal Gene Transfer
Source: PLoS Genet. 2014 Feb 27;10(2):e1004171. doi: 10.1371/journal.pgen.1004171 (PMC3937220; doi:10.1371/journal.pgen.1004171)
Supplement: Figure S5 — Temperature effects on plasmid promoters. Charts show the GFP/OD values achieved in steady-state by the promoters indicated in the figure. Expression profiling was performed as described in Materials and Methods. Cells were grown at 37 C overnight, then diluted 1∶10000 in fresh media, and then grown at the indicated temperatures. (DOCX) [file pgen.1004171.s005.docx]

**Supporting Figure S5. Temperature effects.**
