# Supplementary material for: Negative Feedback and Transcriptional Overshooting in a Regulatory Network for Horizontal Gene Transfer
Source: PLoS Genet. 2014 Feb 27;10(2):e1004171. doi: 10.1371/journal.pgen.1004171 (PMC3937220; doi:10.1371/journal.pgen.1004171)
Supplement: Figure S6 — Presence of potential receptors. Expression profiles of plasmid R388 promoters, obtained as described in Materials and Methods. The effect of potential recipients for horizontal transfer was tested by co-culture with empty E.coli Bw27783. Cells were mixed at 1∶1 ratio before the measurement started. To obtain the same amount of GFP-producing cells, the volume of recipient-containing cultures was doubled. The only effect observed was a general decrease in fluorescence signal in those cultures that contained recipients. Cell quenching probably caused this unspecific effect. (DOCX) [file pgen.1004171.s006.docx]

**Supporting Figure S6 Presence of potential receptors**
