# Supplementary material for: Negative Feedback and Transcriptional Overshooting in a Regulatory Network for Horizontal Gene Transfer
Source: PLoS Genet. 2014 Feb 27;10(2):e1004171. doi: 10.1371/journal.pgen.1004171 (PMC3937220; doi:10.1371/journal.pgen.1004171)
Supplement: Figure S7 — Transient overshooting in StbA/KorA Incoherent Feed Forward Loop (IFFL). In order to test whether the transient overshooting would also happen in more complex architectures apart from simple NFLs, we simulated the behavior of the KorA-StbA IFFL loop present in the conjugation region (Upper panel). The parameters were introduced according to the results depicted in Table S1, which indicate the order of promoter strengths (PstbA>PtrwH>PkorA) and indicated also the relative strengths of repression exerted by the two regulators (KStbA_PkorA >>KKorA_PkorA and KStbA_PtrwH>>KKorA_PtrwH). Results shown in the lower panel indicate that this IFFL architecture will also exhibit a transient overshoot. (DOCX) [file pgen.1004171.s007.docx]

**Supporting Figure S7 Transient overshooting in IFFL loops**
