# Supplementary material for: Negative Feedback and Transcriptional Overshooting in a Regulatory Network for Horizontal Gene Transfer
Source: PLoS Genet. 2014 Feb 27;10(2):e1004171. doi: 10.1371/journal.pgen.1004171 (PMC3937220; doi:10.1371/journal.pgen.1004171)
Supplement: Figure S8 — Growth rate deficit after horizontal gene transfer A. Growth rate after conjugation. (Left panel) Growth rate of Recipients (R), Donors (D) and Transconjugants (T). Cells were mixed at a 1∶1 ratio and allowed to conjugate for 30 min. at 37 C, on LB agar plates. Donors were E.coli Bw27783 Rifr containing plasmid R388, and recipients were E.coli Bw27783 Nxr. Cells were then resuspended in liquid LB and allowed to grow. Cell numbers were obtained by plating on appropriate antibiotic combinations, as indicated in materials and methods. (Right panel) Proportion of plasmid-containing cells that are transconjugants along time. The x axis indicates the timespan since cells were taken out from conjugation mixtures. The y axis indicates the proportion of transconjugants over plasmid-. containing cells (donors + transconjugants). Plasmid R388 does not conjugate in liquid, thus any change in this proportion was due to growth differences. Lower bars indicate the apparent generation times for each species, calculated from the data shown in the left panel. B. Growth rate after mobilization. Growth rate of Recipients (R), Donors (D) and Transconjugants (T). Cells were mixed at a 1∶1 ratio and allowed to conjugate for 30 min at 37 C, on LB agar plates. Donors were E.coli Bw27783 Rifr containing plasmid R388Δnic, and the mobilizable vector pSU4910 (Cmr). R388Δnic encodes for the entire transfer system, but lacks the nic site needed in cis for a DNA to be transferred by conjugation. Thus this strain is able to mobilize pSU4910 without transferring plasmid R388. Recipients were E.coli Bw27783 Nxr. Experiments were performed as in conjugation assays. (DOCX) [file pgen.1004171.s008.docx]

**Supporting Figure S8 Growth rate deficit in early transconjugant**

**A**

**B**
