# Supplementary material for: Negative Feedback and Transcriptional Overshooting in a Regulatory Network for Horizontal Gene Transfer
Source: PLoS Genet. 2014 Feb 27;10(2):e1004171. doi: 10.1371/journal.pgen.1004171 (PMC3937220; doi:10.1371/journal.pgen.1004171)
Supplement: Table S1 — Promoter activities in the presence of plasmid R388 and plasmid transcriptional regulators. (DOCX) [file pgen.1004171.s009.docx]

**Supplementary Table S1. Promoter activities in the presence of plasmid R388 and plasmid transcriptional regulators.**

(*) The table shows steady-state transcription values for plasmid promoters indicated on the rows. E. coli strain BW27783 was transformed with sets of two plasmids: one from the promoter-reporter library (rows) and one from the regulator library (columns). Besides the regulators indicated in the table, the effects of other potential regulators were also tested: *repA*, *orf7*, *orf8*, *orf12*, *orf14* and *korB.* None was shown to have a significant effect on any of the promoters tested (data not shown).

(a) *Open Loop*: Promoter activities obtained when cells contained the promoters and the empty vector pBAD33. Units are in GFP OD ^-1^ t ^-1^ (x10^2^). Time is in minutes.

(b) *Closed Loop*: Promoter activities obtained when cells contained the promoters and plasmid R388. Units are in GFP OD ^-1^ t ^-1^ (x10^2^). Time is in minutes.

(c)Feedback Gain: Ratio between the transcriptional activities of the open loop and the closed loop (Open Loop/Closed Loop). Units are in GFP OD ^-1^ t ^-1^ (x10^2^). Time is in minutes.

(d) Transcriptional Regulators: Promoter activities obtained when cells contained the promoters and the expression vector pBAD33 containing each of the genes indicated in the table. Units are in GFP OD ^-1^ t ^-1^ (x10^2^). Time is in minutes.
