# Supplementary material for: Negative Feedback and Transcriptional Overshooting in a Regulatory Network for Horizontal Gene Transfer
Source: PLoS Genet. 2014 Feb 27;10(2):e1004171. doi: 10.1371/journal.pgen.1004171 (PMC3937220; doi:10.1371/journal.pgen.1004171)
Supplement: Text S1 — Calculations on the Gain-Overshoot relationship. (PDF) [file pgen.1004171.s011.pdf]

Supporting Text S1- Calculations for the Feedback Gain & Overshoot.

Feedback Gain

Suppose a simple feedback system consisting of an mRNA molecule (X) that encodes a transcriptional repressor (Y), able to repress transcription from its own promoter.

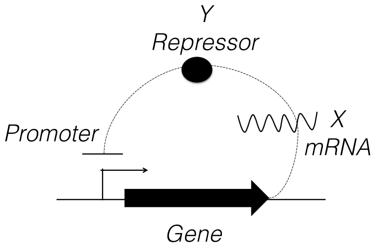

Let G be the feedback gain, defined as the ratio between the steady-states of the negative feedback (X_{fb}) and the open loop (X_0).

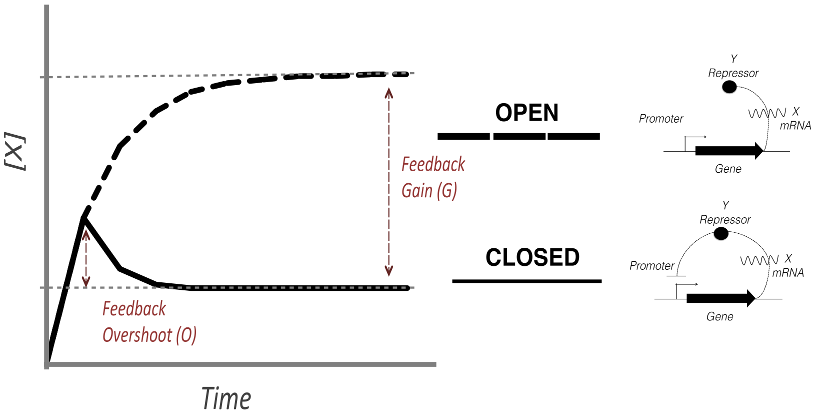

The dynamics of the negative feedback follow

$$\begin{cases} \frac{dx}{dt} = \frac{\lambda_1 k^n}{k^n + (y_t)^n} - \beta_1 x_t \\ \frac{dy}{dt} = \lambda_2 x_t - \beta_2 y_t \end{cases}$$

Eq 1.

Where x is the RNA concentration, Y is the protein concentration , λ stands for production rates, β represents degradation/dilution rates, k is the feedback constant, and n is the cooperativity of the system (non-linearity of the feedback). In these condition the steady-state of the system is

$$\begin{cases} \frac{\text{dx}}{\text{dt}} = \frac{\lambda_1 k^n}{k^n + (y_t)^n} - \beta_1 x_t = 0 \\ \frac{\text{dy}}{\text{dt}} = \lambda_2 x_t - \beta_2 y_t = 0 \end{cases}$$

In order to simplify the calculations we replace $k^n = K$ and the steady-state for strong repression ($K + y_{ss} \simeq y_{ss}$) for X follows

$$X_{fb} = \left(\frac{\lambda_1 K}{\beta_1}\right)^{\frac{1}{n+1}} \left(\frac{\beta_2}{\lambda_2}\right)^{\frac{n}{n+1}}$$

Eq 2.

Similarly the equations for the open loop

$$\begin{cases} \frac{\text{dx}}{\text{dt}} = \lambda_1 - \beta_1 x_t \\ \frac{\text{dy}}{\text{dt}} = \lambda_2 x_t - \beta_2 y_t \end{cases}$$

Eq 3.

yield in steady-state

$$X_0 = \frac{\lambda_1}{\beta_1}$$

Therefore the feedback gain equals

$$\text{G} = \frac{X_o}{X_{fb}} = \left(\frac{\lambda_1 \lambda_2}{\beta_1 \beta_2}\right)^{\frac{n}{n+1}} \left(\frac{1}{K}\right)^{\frac{1}{n+1}} = \left(\frac{\lambda_1 \lambda_2}{\beta_1 \beta_2 k}\right)^{\frac{n}{n+1}}$$

$$\text{G} = \left(\frac{\lambda_1 \lambda_2}{\beta_1 \beta_2 k}\right)^{\frac{n}{n+1}}$$

Eq 4.

This expressions yields the following limits:

$$\underset{k\to 0}{\text{Lim}}\left(\frac{X_o}{X_{fb}} = \left(\frac{\lambda_1 \lambda_2}{\beta_1 \beta_2}\right)^{\frac{n}{n+1}} \left(\frac{1}{K}\right)^{\frac{1}{n+1}}\right) = \underset{k\to 0}{\text{Lim}}\left(\frac{X_o}{X_{fb}} = \left(\frac{\lambda_1 \lambda_2}{\beta_1 \beta_2}\right)^{\frac{n}{n+1}} \left(\frac{1}{k^n}\right)^{\frac{1}{n+1}}\right) = \underset{k\to 0}{\text{Lim}}\left(\frac{X_o}{X_{fb}} = \left(\frac{\lambda_1 \lambda_2}{\beta_1 \beta_2 k}\right)^{\frac{n}{n+1}}\right) = \infty$$

Eq 5.

$$\underset{n\to\infty}{\text{Lim}}\left(\frac{X_o}{X_{fb}} = \left(\frac{\lambda_1 \lambda_2}{\beta_1 \beta_2}\right)^{\frac{n}{n+1}} \left(\frac{1}{K}\right)^{\frac{1}{n+1}}\right) = \underset{n\to\infty}{\text{Lim}}\left(\left(\frac{\lambda_1 \lambda_2}{\beta_1 \beta_2}\right)^{\frac{n}{n+1}} \left(\frac{1}{k^n}\right)^{\frac{1}{n+1}}\right) = \underset{n\to\infty}{\text{Lim}}\left(\frac{\lambda_1 \lambda_2}{\beta_1 \beta_2 k}\right)^{\frac{n}{n+1}} = \left(\frac{\lambda_1 \lambda_2}{\beta_1 \beta_2 k}\right)^1 = \frac{\lambda_1 \lambda_2}{\beta_1 \beta_2 k}$$

Eq 6.

This indicates that while decreasing k (increasing the affinity of the repressor for its cognate site) increases indefinitely the feedback gain, increasing n reaches a limit gain that equals the intrinsic promoter strength ($\frac{\lambda_1 \lambda_2}{\beta_1 \beta_2}$) times the inverse of the half-repression constant (k).

The gain in y is calculated in the same way, yielding

$$\text{G} = \frac{y_o}{y_{fb}} = \left(\frac{\lambda_1 \lambda_2}{\beta_1 \beta_2}\right)^{\frac{n}{n+1}} \left(\frac{1}{K}\right)^{\frac{1}{n+1}} = \left(\frac{\lambda_1 \lambda_2}{\beta_1 \beta_2 k}\right)^{\frac{n}{n+1}}$$

$$\text{G} = \frac{y_o}{y_{fb}} = \left(\frac{\lambda_1 \lambda_2}{\beta_1 \beta_2 k}\right)^{\frac{n}{n+1}}$$

Which indicates that the feedback gain is equivalent for values of x and y

Feedback Overshoot existence

System of Ec. 1 produces a transient overshoot whenever x and y reach a maximum that is higher than the steady state value. A max. in x is reached iif

$$x' = \frac{dx}{dt} = 0 \text{ and } x'' = \frac{d^2 x}{dt} < 0$$

$$\frac{dx}{dt} = \frac{\lambda_1 K}{K + (y_t)^n} - \beta_1 x_{max} = 0$$

$$x_{max} = \frac{\lambda_1 K}{\beta_1 (K + (y_t)^n} - \frac{x'}{\beta_1} = \frac{\lambda_1 K}{\beta_1 (K + (y_t)^n}$$

$$x'' = \lambda_1 K \frac{0 - n\, y^{n-1} y'}{(K + y^n)^2} = \lambda_1 K \frac{-n\, y^{n-1} y'}{(K + y^n)^2}$$

$$x'' = \lambda_1 K \frac{-n}{y^{n+1}} y' < 0$$

$-n < 0$ since there cannot be negative cooperativity
$y^{n+1} > 0$ since y cannot take negative values
λ_1 K are always positive

then $x'' < 0 \longleftrightarrow y' > 0$

From this follows that y at $x = x_{max}$ y is always smaller than its steady state value

$$y' = \lambda_2 x - \beta_2 y$$

$$y' = \lambda_2 x - \beta_2 y_{x_{max}}$$

$$\frac{\lambda_1 \lambda_2 K}{\beta_{1\beta}} > y_{x_{max}}{}^{n+1}$$

Since $y_{ss} = \left(\frac{\lambda_1 \lambda_2 K}{\beta_{1\beta}}\right)^{\frac{1}{n+1}}$

$$\left(\frac{\lambda_1 \lambda_2 K}{\beta_{1\beta}}\right)^{\frac{1}{n+1}} > y_{x_{max}}$$

$$y_{ss} > y_{x_{max}}$$

Equivalent reasoning yields that $x' < 0$ at $y = y_{max}$ and $x_{ss} < x_{y_{max}}$

Gain-Overshoot relationship

The overshoot (O) is the ratio between the maximal value of X or Y and its steady-state value. In the case of an RNA

$$O = \frac{X_{max}}{Xss} = \frac{X_{max}}{\left(\frac{\lambda_1 K}{\beta_1}\right)^{\frac{1}{n+1}} \left(\frac{\beta_2}{\lambda_2}\right)^{\frac{n}{n+1}}} = \frac{X_{max}}{\left(\frac{\lambda_1}{\beta_1}\right)^{\frac{1}{n+1}} \left(\frac{\beta_2}{\lambda_2} k\right)^{\frac{n}{n+1}}} \qquad \text{Eq.7}$$

The higly non-linear nature of the ODE system prevents the calculation of Xmax, but in the case of strong self-repression (k+y ≈ y) and high cooperativity we can linearize the system, approximating Xmax to the equivalent

value for the open loop when t= $t_{x_{\max}}$

$\frac{dx}{dt} = \lambda_1 - \beta_1 x_t$ from t=0 $\longrightarrow t = t_{x=\text{xmax}}$

$\frac{dx}{dt} = \frac{\lambda_1 K}{K + (y_t)^n} - \beta_1 x_t$ from $t = t_{x=\text{xmax}} \longrightarrow t = \infty$

This allows direct integration of Xmax

$$\int \frac{dx}{dt} \, dt = \int_0^{t=t_{x=\text{xmax}}} (\lambda_1 - \beta_1 x_t) \, dt = \frac{\lambda_1}{\beta_1} \left(1 - e^{-\beta_1 t_{x=x_{\max}}}\right)$$

This approximation holds for higly-nolinear systems, where the high cooperativity index (n) acts as an effective delay between the accumulation of the repressor (y) and the onset of repression. Therefore

$$O_x = \frac{X_{\max}}{\text{Xss}} \approx \frac{\frac{\lambda_1}{\beta_1}\left(1 - e^{-\beta_1 t_{x=x_{\max}}}\right)}{\left(\frac{\lambda_1 K}{\beta_1}\right)^{\frac{1}{n+1}} \left(\frac{\beta_2}{\lambda_2}\right)^{\frac{n}{n+1}}} = \left(\frac{\lambda_1 \lambda_2}{\beta_1 \beta_2}\right)^{\frac{n}{n+1}} \left(\frac{1}{K}\right)^{\frac{1}{n+1}} \left(1 - e^{-\beta_1 t_{x=x_{\max}}}\right)$$

$$O_x \approx \left(1 - e^{-\beta_1 t_{x=x_{\max}}}\right) \text{G}$$

To calculate the overshoot for y we follow the same reasoning. Linearizing x until the onset of the negative feedback, we can approximate the value of Ymax

$$O_x = \frac{Y_{\max}}{\text{Yss}}$$

$$\frac{dy}{dt} = \lambda_2 x_t - \beta_2 y_t \text{ and } x_t \approx \frac{\lambda_1}{\beta_1} \left(1 - e^{-\beta_1 t}\right)$$

$$\frac{dy}{dt} \approx \frac{\lambda_1 \lambda_2}{\beta_1} \left(1 - e^{-\beta_1 t_{x=x_{\max}}}\right) - \beta_2 y_t$$

$$O_y \approx \left(\left(\frac{\lambda_1 \lambda_2}{\beta_1 \beta_2}\right)^{\frac{n}{n+1}} \left(\frac{1}{K}\right)^{\frac{1}{n+1}}\right) \left(\frac{\beta_1\left(1 - e^{-\beta_1 t}\right) - \beta_2\left(1 - e^{-\beta_2 t}\right)}{\beta_1 - \beta_2}\right)$$

$$O_y \approx \left(\frac{\beta_1\left(1 - e^{-\beta_1 t}\right) - \beta_2\left(1 - e^{-\beta_2 t}\right)}{\beta_1 - \beta_2}\right) \text{G}$$

This indicates that the $O_y \neq O_x$, and

$$O_y = O_x \left(\frac{\beta_1 - \beta_2 \frac{\left(1 - e^{-\beta_2 t}\right)}{\left(1 - e^{-\beta_1 t}\right)}}{\beta_1 - \beta_2}\right)$$

Simulations indicate that these approximation holds for highly-nonlinear systems (Figure 4, main text). This condition can be met by systems where the repressor exhibits high cooperativity to its cognate binding site or where the repressor dimer/multimerization is required for binding.

Effect of multimerization

The following system includes a step of repressor dimerization

$(k^n = K)$

$\frac{dx}{dt} = \frac{\lambda_1 K}{K + (z_t)^n} - \beta_1 x_t$

$\frac{dy}{dt} = \lambda_2 x_t - \beta_2 y_t$

$\frac{dz}{dt} = c_a y - c_d z$

And in steady-state

$\frac{dz}{dt} = c_a y - c_d z$

$z = \frac{c_a}{c_d} y$

Therfore

$\frac{dx}{dt} = \frac{\lambda_1 K}{K + (z_{ss})^n} - \beta_1 x_{ss} = \frac{\lambda_1 K}{K + \left(\frac{c_a}{c_d} y_{ss}\right)^n} - \beta_1 x_{ss} = 0$

This indicates that at steady state the dimer system is formally identical to the monomeric system with the only difference of c_a/c_d multiplying y, which reflects the steady-state of the dimerization dynamics. Similarly, the gain in the dimer system can be expressed as

$$G = \frac{X_o}{X_{fb}} = \left(\frac{\lambda_1 \lambda_2 \frac{c_a}{c_d}}{\beta_1 \beta_2 k}\right)^{\frac{n}{n+1}}$$
